# Counting the Countless: Bacterial Quantification by Targeting rRNA Molecules to Explore the Human Gut Microbiota in Health and Disease

**DOI:** 10.3389/fmicb.2018.01417

**Published:** 2018-06-29

**Authors:** Hirokazu Tsuji, Kazunori Matsuda, Koji Nomoto

**Affiliations:** ^1^Basic Research Department, Yakult Central Institute, Tokyo, Japan; ^2^Yakult Honsha European Research Center for Microbiology ESV, Gent-Zwijnaarde, Belgium

**Keywords:** age-related bacterial community, bacterial quantification, disease-specific dysbiosis, gut microbiota, RT-qPCR, YIF-SCAN

## Abstract

Over the past decade, the advent of next-generation-sequencing tools has revolutionized our approach to understanding the human gut microbiota. However, numerical data on the gut bacterial groups—particularly low-cell-count microbiota, such as indigenous pathobionts, that are otherwise important components of the microbiota—are relatively limited and disparate. As a result, the comprehensive quantitative structure of the human gut microbiota still needs to be fully defined and standardized. With the aim of filling this knowledge gap, we have established a highly sensitive quantitative analytical system that is based on reverse transcription—quantitative PCR and targets microbial rRNA molecules. The system has already been validated in the precise, sensitive, and absolute quantification of more than 70 target bacterial groups belonging to various human gut bacterial clades, including predominant obligate and facultative anaerobes. The system demonstrates sensitivity several hundred times greater than that of other rRNA-gene-targeting methods. It is thus an efficient and valuable tool for exhaustive analysis of gut microbiota over a wide dynamic range. Using this system, we have to date quantified the gut microbiota of about 2,000 healthy Japanese subjects ranging in age from 1 day to over 80 years. By integrating and analyzing this large database, we came across several novel and interesting features of the gut microbiota, which we discuss here. For instance, we demonstrated for the first time that the fecal counts of not only the predominant bacterial groups but also those at lower cell counts conform to a logarithmically normal distribution. In addition, we revealed several interesting quantitative differences in the gut microbiota of people from different age groups and countries and with different diseases. Because of its high analytic sensitivity, the system has also been applied successfully to other body niches, such as in characterizing the vaginal microbiota, detecting septicemia, and monitoring bacterial translocation. Here, we present a quantitative perspective on the human gut microbiota and review some of the novel microbial insights revealed by employing this promising analytical approach.

## Introduction

The human intestine harbors a highly complex microbial ecosystem (Turnbaugh et al., 2007) comprising as many as 1,000 different kinds of bacteria, collectively known as the intestinal microbiota, which plays a fundamental role in human health (Turnbaugh et al., 2006; Round and Mazmanian, 2009; Arumugam et al., 2011; Cho and Blaser, 2012; Yatsunenko et al., 2012; Koeth et al., 2013; Yoshimoto et al., 2013). The intestinal microbiota consists of various microbial communities that are present at different microbial cell counts and can be categorized as (a) predominant strictly anaerobic bacterial groups such as *Clostridium coccoides* group, *Clostridium leptum* subgroup, *Bacteroides fragilis* group and *Bifidobacterium*, (b) low-cell-count microbiota (LCCM) including facultative anaerobic groups such as *Enterobacteriaceae, Lactobacillus, Enterococcus*, and *Staphylococcus*, and (c) low-population-level indigenous pathobionts such as *Clostridium difficile* and *Clostridium perfringens* at various population densities (Ozaki et al., 2004; Tonooka et al., 2005; Lakshminarayanan et al., 2013). The cell counts of these diverse bacterial groups are highly controlled by “colonization resistance,” which is a means by which the microbiota and the host maintain a stable balance (van der Waaij et al., 1971; Buffie and Pamer, 2013; Lawley et al., 2013).

Under normal conditions, the healthy intestinal microbiota exists in homeostasis with the host, whereby the population growth (and toxigenicity) of LCCM including pathobionts remains controlled. However, in a state of dysbiosis (i.e., intestinal microbial imbalance), these LCCM can multiply and cause serious problems in clinical settings (Gorkiewicz, 2009; Litvak et al., 2017; Samarkos et al., 2018). Therefore, to comprehensively assess the intestinal microbiota—a diverse microbial ecosystem with a wide dynamic range—it is essential that we precisely understand not only the relative abundance, but also the microbial cell counts of the predominant bacterial groups and the LCCM—including facultative anaerobes and indigenous pathobionts. Accordingly, even in this golden age of advanced sequencing tools, quantitative data corresponding to the “gold standard” viable bacterial count remain critically important.

Recently, Vandeputte et al. (2017) constructed a workflow for the quantitative microbiome profiling of fecal bacteria combining 16S rRNA gene PCR amplicon sequencing and flow cytometric quantification of microbial cells, and demonstrated that the microbial loads of healthy individuals can vary across a 10-fold dynamic range and also that microbial loads are a key driver for microbiota alterations in patients with Crohn's disease. The authors stated: “To enable genuine characterization of host-microbiota interactions, microbiome research must exchange ratios for counts” (Vandeputte et al., 2017). To address this, we have developed a sensitive and absolute quantitative analytical system (known in-house as Yakult-Intestinal-Flora-Scan; hereafter referred to as YIF-SCAN) that is based on reverse transcription—quantitative PCR (RT-qPCR) and targets rRNA molecules abundant in microbes (Matsuda et al., 2007, 2009). YIF-SCAN quantifies the predominant human intestinal bacteria at a sensitivity equivalent to that of qPCR or fluorescent *in-situ* hybridization (FISH) methods (detection limit: 10^5^ to 10^8^ cells/g feces); LCCM can be enumerated at sensitivities comparable to those of culture methods (detection limit: 10^2^ to 10^3^ cells/g feces: ~100 to 1,000 times greater than that of qPCR and 100,000 to 1,000,000 times greater than that of FISH) (Matsuda et al., 2007, 2009, 2012; Kubota et al., 2010; Kurakawa et al., 2012, 2013).

Using this analytical system, we have to date analyzed and reported the quantitative gut microbial composition of more than 1,900 healthy Japanese subjects ranging in age from 1 day to over 80 years (Nomoto et al., 2015). Recently, this database was further augmented by the addition of 451 healthy subjects (Hasegawa et al., 2015; Morita et al., 2015; Nakayama et al., 2015; Aizawa et al., 2016; Suzuki et al., 2017) and analyzed using several newly validated bacterial assays that were not previously executed. Taking into account the hypotheses and preliminary data from our previous investigations, we subjected this database to additional exhaustive statistical analyses that revealed some intriguing features of the gut microbiota. For instance, we demonstrate that fecal counts of not only the predominant obligate anaerobes but also the facultative anaerobes follow a logarithmic normal distribution. *Prevotella*, however, emerges as a surprising exception: it exhibits a multimodal distribution pattern. Interestingly, several age-dependent, gender-specific differences in intestinal microbial composition are also revealed by this intensive analysis. Furthermore, by integrating this database and using it as a reference, we reveal some important features of gut dysbiosis in several diseases and demonstrate regional differences in the intestinal microbiota among several Asian or Oceanic populations. YIF-SCAN analysis has also enabled us to precisely monitor the gut microbial changes that occur following the ingestion of probiotics or synbiotics. Given that quantitative data are likely to prove invaluable for understanding the roles of different gut microbes in health and disease, the high sensitivity of YIF-SCAN makes it a very useful tool in both gut microbiota exploration and microbial diagnosis in clinical practice.

## Targeting rRNA molecules for bacterial quantification: a new frontier in gut microbiota analysis

As mentioned above, YIF-SCAN is based on RT-qPCR. Generally, bacteria contain thousands to tens of thousands of ribosomes, which include rRNA molecules that can be classified into 5S, 16S, and 23S subunits according to their sedimentation rate. However, only a few to a dozen copies of rRNA genes are encoded on the bacterial chromosome. Although FISH has been used to quantify bacteria by targeting rRNA molecules, the detection sensitivity of this method is only at the level of 10^8^ cells/g feces (Matsuki et al., 2004a). We speculated that quantitative analysis targeting rRNA molecules would greatly improve analytical sensitivity. When primers targeting the same rRNA gene sequence are used, there is no difference in the slope of the standard curve between qPCR and RT-qPCR, but the difference in the y-intercept (threshold cycle [Ct] value) reveals at least 100 times increased sensitivity (Matsuda et al., 2007, 2012; Kubota et al., 2010; Kurakawa et al., 2012; Ogata et al., 2015). Furthermore, rRNA-gene-based PCR often measures not only viable bacteria but also free bacterial DNA or the DNA from dead bacteria, whereas the detection of rRNA molecules by RT-qPCR is more closely associated with bacterial viability. In fact, studies using *in vitro* cultures have shown that, for several different bacterial strains, YIF-SCAN data corresponds to that obtained by the culture method throughout the growth phases and during the process of cell death (Matsuda et al., 2007; Kurakawa et al., 2012). RT-qPCR quantification of rRNA molecules thereby provides a combination of both cell abundance and viability, and is therefore an efficient and preferable method for quantifying live intestinal bacteria.

It should be noted that certain bacteria may remain underrepresented or underestimated in DNA-sequencing-based datasets because of technical anomalies such as a bias of PCR primers against particular 16S rRNA gene sequences or the use of DNA extraction methods that are not equally effective for all gut inhabitants (e.g., high vs. low G+C, or Gram positive vs. negative). Also, although sequencing methods generate tens of thousands of rRNA gene sequences per DNA sample, there could be several hundred operational taxonomic units in a given sample and hence it is possible that taxa with very low abundance could be overlooked (Tannock et al., 2016). Moreover, given that only a fragment of the rRNA gene is sequenced, the resolution may not be adequate and consistent enough for species-level discrimination. Given that it is challenging to design a universal primer that amplifies the gene sequences of all gut microbes with equal efficiency, to achieve detailed information it is necessary to enumerate bacterial groups by using specific as well as sensitive primer sets. To this end, YIF-SCAN uses thoroughly validated specific primers sets and targets 16S rRNA molecules, which are present at huge copy numbers per cell (compared with gene copy numbers), thereby yielding high analytical sensitivity and precise and absolute quantification data (Matsuda et al., 2007, 2009). The YIF-SCAN analysis consists of the following operations: (1) collection and storage of feces, (2) isolation of fecal RNA, (3) preparation of reaction microplate for PCR assay, (4) RT-qPCR, and (5) calculation of bacterial count from PCR data. In order to automate the series of the operations of No. 2, 3, and 4, we have installed the following hardware: a liquid handling workstation, a robotic centrifuge, a whole rack tube capper/decapper, and a robotic handling machine. These hardware are connected with real-time PCR devices via the robotic handling machine, and the integrated system is automatically managed by the respective drivers and a scheduling software. In this system, the fecal samples are dispensed into 96 whole rack tubes and are controlled using barcode technology. The automation system has dramatically improved the throughput and reproducibility of YIF-SCAN, and has decreased the risks that researchers might be contaminated by fecal bacteria and chemicals during the RNA extracting operations.

For YIF-SCAN analysis, we established a pool of more than 70 primer sets and the corresponding nucleic acids (RNA) from standard strains of various gut microbiota (Table 1). A strain representing a target bacterial group was used as the standard strain. RNA was extracted from each standard strain by using the same method as for fecal samples in order to delete experimental biases associated with differences in amplification efficiency among primer sets and differences in RNA extraction efficiency among bacterial species. Using a pool of primer sets facilitated the quantification of a wide range of subgroups or species belonging to various human gut bacterial clades, including predominant obligate anaerobes, facultative anaerobes (as well as LCCM), and opportunistic or potential pathogens (Table 1). Notably, all of the primer sets had been thoroughly validated for specific, sensitive, and absolute quantification of the corresponding targets in human feces. In addition to the major predominant obligate anaerobes, this in-house validated pool encompassed facultative anaerobes present as LCCM in the intestine, thereby enabling an exhaustive analysis of gut microbiota across a wide dynamic range. For example, in the case of *Lactobacillus*, eight subgroups and species can be analyzed, whereas in the case of *Bifidobacterium* eight bacterial species commonly detected in humans can be examined. Detection systems for species that belong to the Gram-positive catalase-negative cocci, such as *Streptococcus, Enterococcus*, and *Lactococcus* which are prevalent in the human intestine have also been developed (Kubota et al., 2010). Furthermore, we also analyzed intestinal pathogens such as *Vibrio cholerae*/*mimicus, Vibrio parahaemolyticus*, and *Campylobacter* (Kurakawa et al., 2012; Nair et al., 2012), as well as several other opportunistic pathogens and fungi (Matsuda et al., 2012; Nagpal et al., 2015; Ogata et al., 2015).

**Table 1:** List of bacterial groups included in the YIF-SCAN analytical system.

**Bacterial groups**	**Detection limit (log_10_ cells/g feces)**	**References for primer sets**
**OBLIGATE ANAEROBES**		
*Clostridium coccoides* group	4	Matsuki et al., 2002
*Clostridium hathewayi*	4	Kurakawa et al., 2015a
*Clostridium indolis* subgroup	3	
*Clostridium symbiosum*	4	
*Fusicatenibacter saccharivorans*	3	
Genus *Blautia*	4	
*Clostridium nexile*	3	
*Ruminococcus gnavus*	4	
*Anaerostipes caccae*	3	
*Ruminococcus lactaris*	4	
*Eubacterium ramulus*	3	
*Eubacterium rectale*	3	
*Eubacterium eligens*	4	
*Eubacterium ventriosum*	3	
*Eubacterium hallii*	3	
*Clostridium asparagiforme*	3	
*Coprococcus comes*	2	
*Coprococcus eutactus*	3	
*Roseburia intestinalis*	3	
*Clostridium hylemonae*	4	
*Clostridium scindens*	3	
Genus *Dorea*	3	
*Ruminococcus torques*	3	
*Clostridium leptum* subgroup	4	
*Bacteroides fragilis* group	4	Matsuki, 2007
Genus *Bifidobacterium*	4	Matsuki et al., 1998
*Bifidobacterium adolescentis*	2	Kurakawa et al., 2015b
*Bifidobacterium angulatum*	3	Matsuki et al., 2004a
*Bifidobacterium bifidum*	3	
*Bifidobacterium breve*	3	
*Bifidobacterium catenulatum* group	2	
*Bifidobacterium dentium*	3	
*Bifidobacterium longum* subsp*. longum*	3	Kurakawa et al., 2015b
*Bifidobacterium longum* subsp*. infantis*	3	
*Atopobium* cluster	4	Matsuki et al., 2004b
Genus *Prevotella*	4	Matsuki et al., 2002
*Clostridium perfringens*	2	Matsuda et al., 2009
*Clostridium difficile*	2	Matsuda et al., 2012
**FACULTATIVE ANAEROBES**		
Family *Enterobacteriaceae*	4	Matsuda et al., 2007
*Escherichia coli*	4	Kurakawa et al., 2013
Genus *Lactobacillus*		
*Lactobacillus casei* subgroup	3	Matsuda et al., 2009
*Lactobacillus gasseri* subgroup	3	
*Lactobacillus plantarum* subgroup	3	
*Lactobacillus reuteri* subgroup	3	
*Lactobacillus ruminis* subgroup	3	
*Lactobacillus sakei* subgroup	3	
*Lactobacillus brevis*	3	
*Lactobacillus fermentum*	5	
*Lactobacillus fructivorans*	3	
Genus Enterococcus	3	
*Enterococcus faecalis*	3	Kubota et al., 2010
*Enterococcus caccae*	3	
*Enterococcus cecorum*	4	
*Enterococcus sulfureus* subgroup	4	
*Enterococcus casseliflavus* subgroup	3	
*Enterococcus avium* subgroup	3	
*Enterococcus dispar*	3	
*Enterococcus faecium* subgroup	4	
*Enterococcus faecium*	4	
Genus *Streptococcus*	4	Sakaguchi et al., 2010
*Streptococcus agalactiae*	4	
*Streptococcus pyogenes*	4	
*Streptococcus pneumonia/ mitis*	4	
*Streptococcus salivarius/ thermophiles*	4	Kubota et al., 2010
*Lactococcus lactis* subgroup	4	
*Lactococcus piscium* subgroup	4	
Genus *Staphylococcus*	3	Matsuda et al., 2009
**PATHOGENS**		
Genus *Pseudomonas*	4	Matsuda et al., 2007
*Vibrio cholerae/mimicus*	3	Kurakawa et al., 2012
*Vibrio parahaemolyticus/alginolyticus*	3	
*Campylobacter jejuni/coli*	3	
*Candida* group	2	Ogata et al., 2015
*Candida albicans*	3	
*Candida glabrata*	2	
*Candida krusei*	2	
*Candida tropicalis*	2	
*Candida parapsilosis*	2	

In subsequent sections, we demonstrate some interesting features of the human gut microbiota that have been revealed with the use of this quantitative analytical system. We also introduce the potential application of the system in the clinical diagnosis of bacteremia.

## Quantitative dynamics of the human gut microbiota: insights from microbiota analysis in healthy japanese populations

The last decade has seen remarkable advances in elucidating the intimate relationship between the intestinal microbiota and aging (Hopkins et al., 2001; Woodmansey, 2007; Enck et al., 2009; Biagi et al., 2010; Tiihonen et al., 2010; Claesson et al., 2012; O'Toole, 2012; Yatsunenko et al., 2012; Rampelli et al., 2013; Odamaki et al., 2016). YIF-SCAN can quantify gut bacteria with reproducibility, thereby enabling us to easily and precisely integrate the data from many independent clinical studies and scrutinize the age-related dynamics of the microbiota. We used an integrated database of 11 major human intestinal bacterial genera, and species that have been quantified in different studies in a total of 1951 healthy Japanese subjects (age range: 1 day to 102 years; Supplementary Table 1) and used YIF-SCAN to provide a quantitative profiling of the major human gut bacterial groups of the gut microbiota in the context of host age (Matsuda et al., 2009; Bian et al., 2011; Nagata et al., 2011, 2016; Tsuji et al., 2012, 2014; Ohigashi et al., 2013; Aoki et al., 2014; Sato et al., 2014; Hasegawa et al., 2015; Morita et al., 2015; Nakayama et al., 2015; Wang et al., 2015; Aizawa et al., 2016).

**Figure 1:**
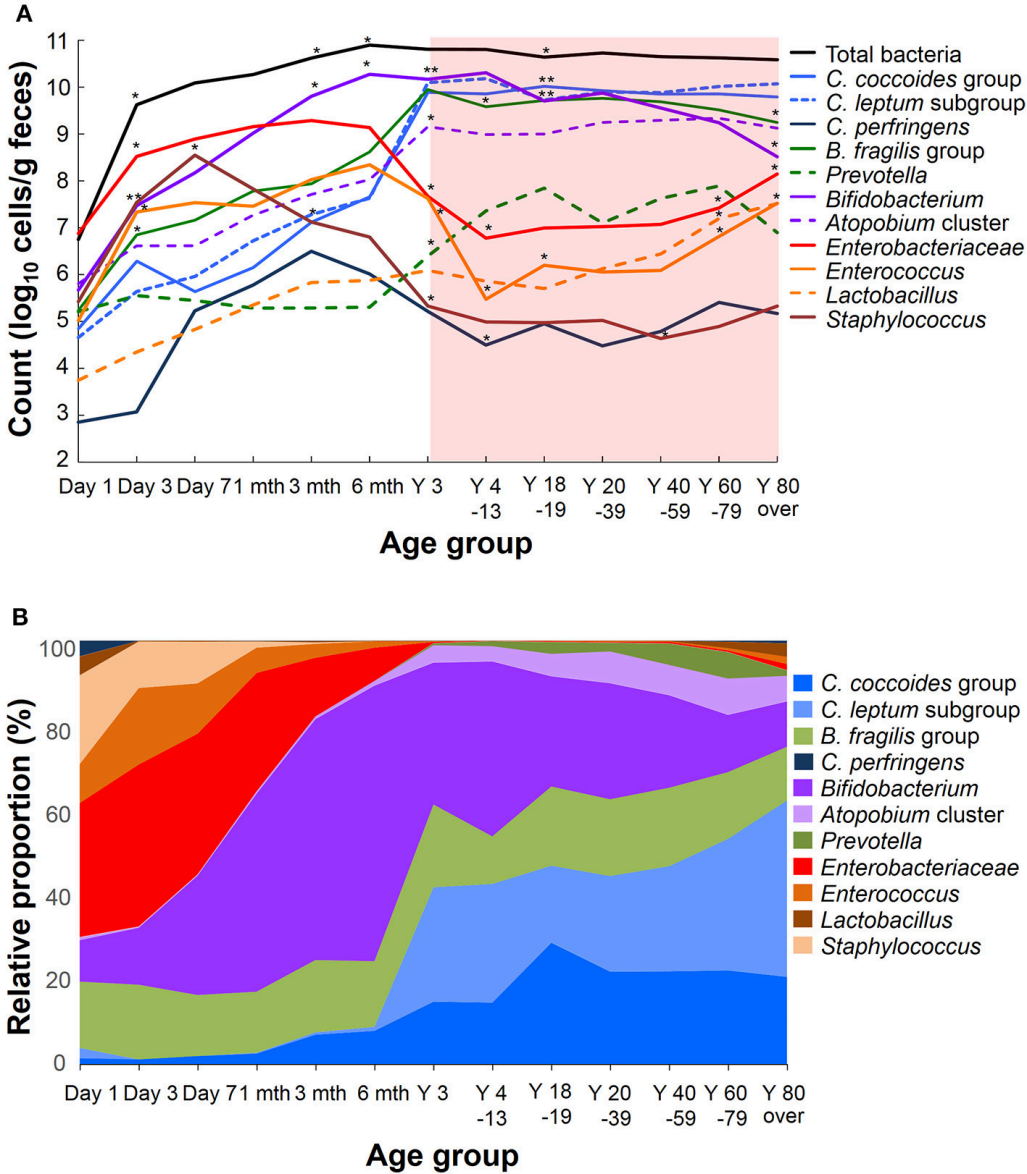
Age-related differences in average counts **(A)** and relative proportions **(B)** of intestinal bacteria in healthy Japanese volunteers (*n* = 1951). An asterisk shows the significant difference between the count of a period and the preceding one (**A**, Steel-Dwass multiple comparison test, ^*^P < 0.05, ^**^P < 0.01).

**Figure 2:**
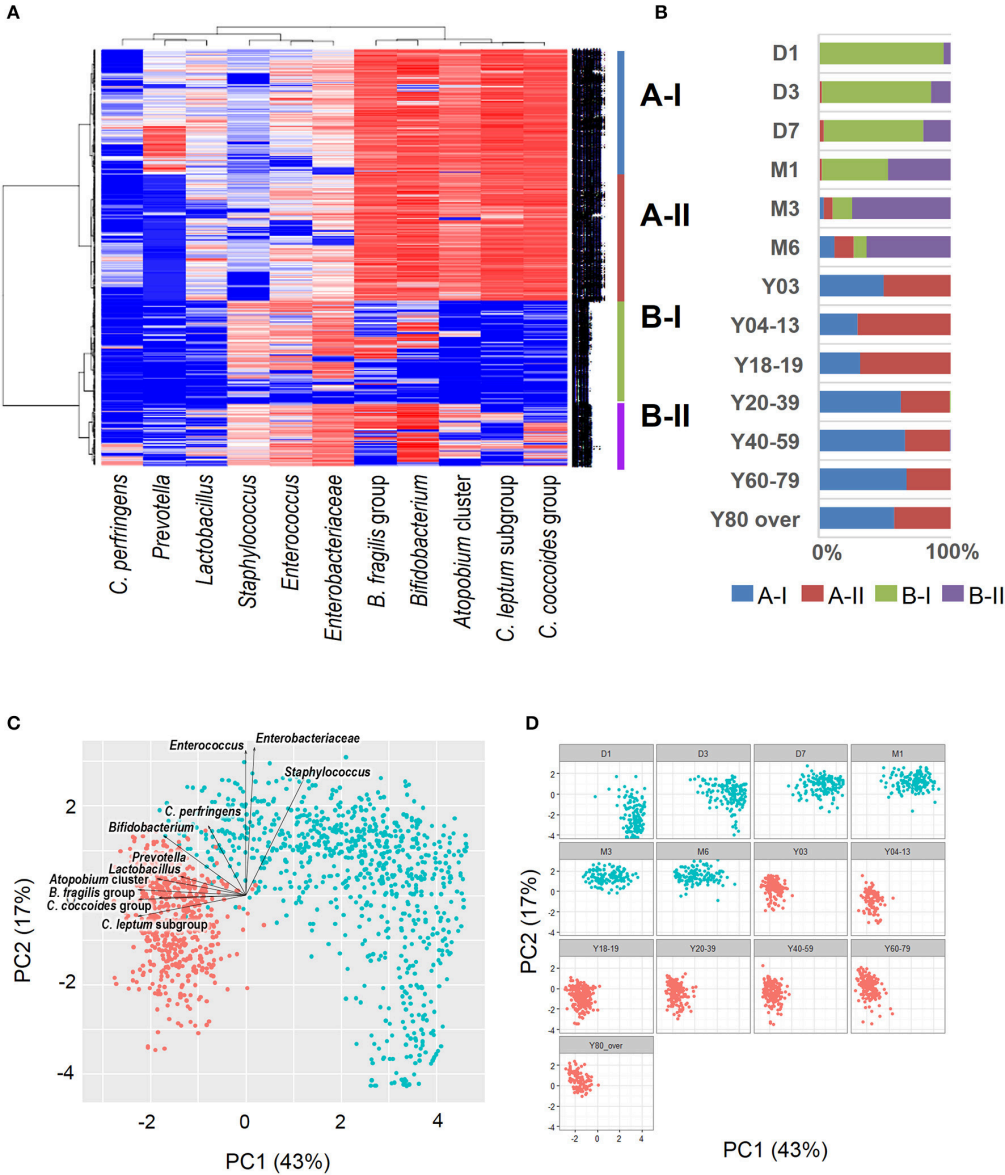
Heat-map analysis **(A)**, classification by hierarchical clustering **(B)**, and principal component analysis plots (**C**: all subjects; **D**: age-wise) of intestinal bacterial microbiota in healthy Japanese volunteers (*n* = 1951). Red dots indicate infant type of microbiota (day 0 to age 6 months) and blue dots indicate adult type (3 years to over 80 years). Arrows indicate characteristic vectors of the factor loadings of 11 bacterial groups.

Recent sequence analyses using bacterial 16S rRNA genes have reported that gut microbiota diversity in newborns undergoes a gradual increase early in life, but reaches a plateau by 3 years of age, at which time the microbial configuration and diversity become equivalent to those in adults (Nakayama, 2010; Yatsunenko et al., 2012; Odamaki et al., 2016). Further age-related quantitative changes in intestinal bacterial populations have been shown by YIF-SCAN analysis the predominant bacterial populations and those present at lower levels, such as *Enterobacteriaceae*, lactobacilli, and pathobionts. Moreover, the data from our integrated database, namely bacterial counts and relative proportions (Figure 1) and heat-map and principal component (PCA) analyses (Figure 2), demonstrate that the intestinal microbiota stabilizes at about age 3 years. However, detailed observation of major 11 intestinal bacterial groups (Figure 1A) reveals that, although the numbers of predominant obligate anaerobes stabilize by age 3 years, several LCCM, including *Prevotella, Enterobacteriaceae*, and *Enterococcus*, are still in the process of transition have not yet stabilized. Also, as reported in previous studies (Hayashi et al., 2003; Bartosch et al., 2004; Woodmansey et al., 2004; Mitsuoka, 2005; Mueller et al., 2006; Tiihonen et al., 2008; Enck et al., 2009; Mariat et al., 2009), in elderly people the levels of *Bifidobacterium* are decreased, whereas those of *Lactobacillus* and *Enterobacteriaceae* are increased (Figures 1A,B). On the basis of hierarchical clustering of the dataset, we were able to classify the 1951 subjects into four groups, namely AI, AII, BI, and BII (Figure 2A); 94.8% of subjects belonging to groups AI and AII were aged 3 years or older (adult type), whereas 99.7% of subjects from groups BI and BII were aged 6 months or less (infant group) (Figure 2B). Notably, these age-related differences are further clearly corroborated by differences in the PCA components of infant-type vs. adult-type cohorts (Figures 2C,D).

**Figure 3:**
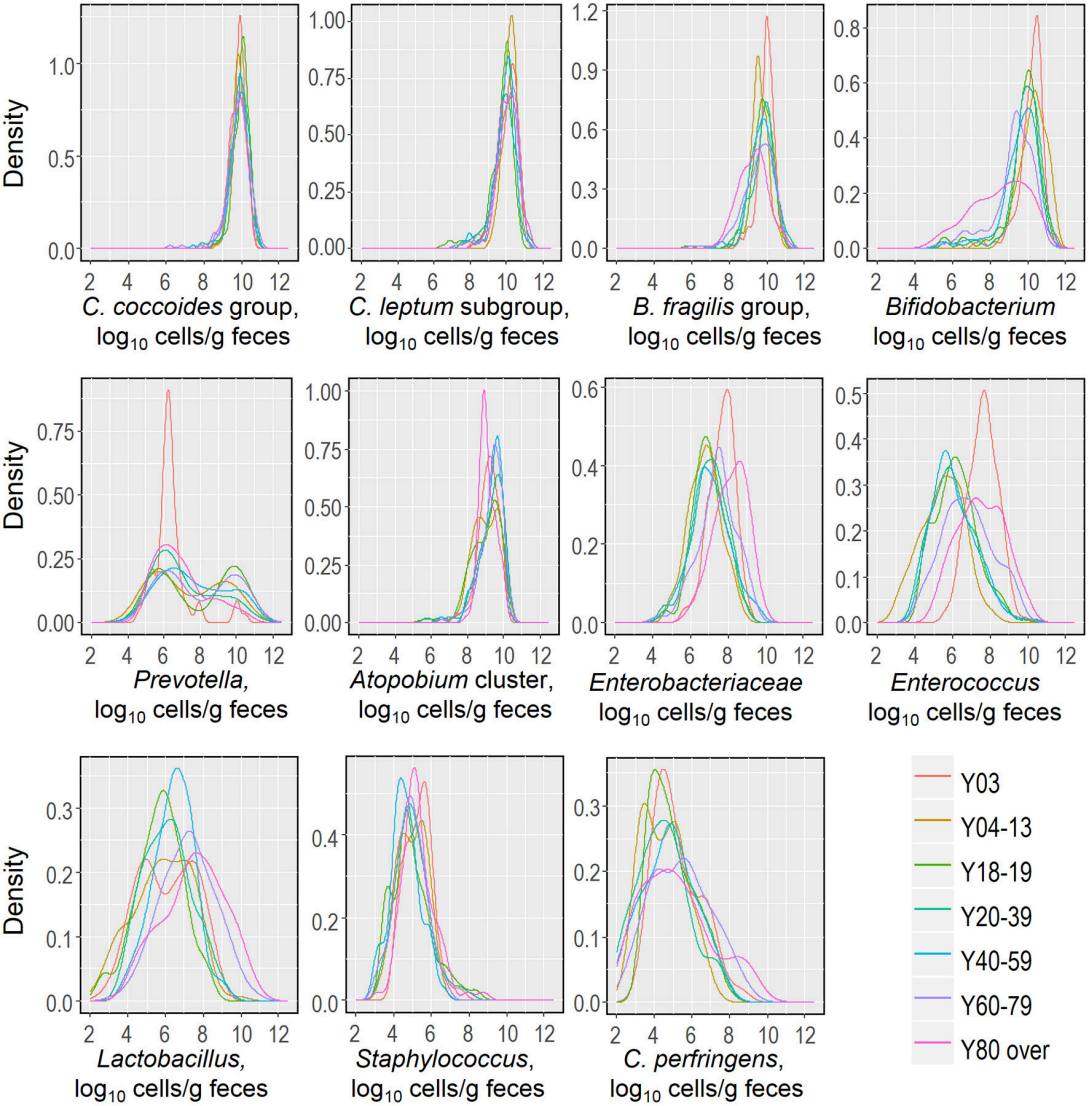
Distributions of fecal counts of major intestinal bacterial groups in healthy Japanese subjects aged 3 years or older (*n* = 1116).

Recently, we reported that the counts of predominant obligate anaerobes (*C. coccoides* group, *C. leptum* subgroup, *B. fragilis* group and *Bifidobacterium*) and low-cell-count facultative anaerobes (*Enterobacteriaceae, Staphylococcus*, and *Lactobacillus*) follow an approximately logarithmic normal distribution in a cohort of healthy Japanese volunteers aged 3 to 102 years (Nomoto et al., 2015), and this observation prompted us to further analyze and estimate this distribution in relation to host age. We created kernel density estimation plots depicting the distribution of bacterial counts in subjects aged 3 years and older (Figure 3). By using Hartigan's dip test statistics, we found that the distributions of almost all major bacterial groups exhibited a unimodal pattern, which could be considered to be an approximately log-normal distribution. However, *Prevotella* seems to be an exception: its distribution exhibited a significant multimodality, particularly in subjects aged 18 to 19 and 60 to 79 years (Table 2). Some specific intestinal bacteria (*B. fragilis* group, *Dialister* spp., *Prevotella* group, uncultured Clostridiales UCI, and UCII) have previously been reported to exhibit bimodal abundance distributions and to form stable states at certain abundance levels associated with host factors such as age and obesity (Lahti et al., 2014). This might corroborate our data on the *Prevotella* distribution in adults. The reason for the multimodal distribution of *Prevotella* is unclear, but plausible causes are diet, age, sex, and genetic factor differences in adulthood. Alternatively, several *Prevotella* spp. may exist simply following the rule of multiple stable configurations in the intestine (Gonze et al., 2017). Both Hartigan's dip test (Table 2) and the histogram peaks (Figure 3) clearly demonstrated that all the major gut bacterial clades—except *Prevotella—*followed an approximately logarithmic normal distribution. Shade et al. (2012) reported that microbial community stability includes resistance and resilience responses to press or pulse disturbances, or both; our result and the above mentioned previous result (Lahti et al., 2014) suggest that the logarithmic normal distribution of bacterial counts indicates that gut bacterial communities are in a state of equilibrium, and that this may be an indicator of a subject's health.

In addition to defining a quantitative view of the core microbiota and the major gut bacterial taxa, YIF-SCAN enables a more detailed analysis of these bacterial groups. For example, in a systematic and quantitative molecular phylogenetic analysis based on the 16S rRNA gene sequence, we classified the *C. coccoides* group (clostridial cluster XIVa = *Lachnospiraceae*), which consist of many bacterial genera or species with a wide variety of functions, into 19 species and three genetically similar subgroups, and specific primers were newly developed for each (Kurakawa et al., 2015a). Further analysis of these taxa in infants, adults, and elderly subjects revealed that (a) the genus *Blautia* and the species *Ruminococcus gnavus* predominated in all age groups, and (b) the genus *Dorea* and the species *Fusicatenibacter saccharivorans* were present in high-cell-counts in adults, whereas (c) *Clostridium scindens* (a secondary bile-acid-producing bacterium) prevailed in the elderly. Various factors, such as diet and lifestyle, have an impact on differences in the predominant anaerobes among age groups (Brown et al., 2012; Clarke et al., 2014; David et al., 2014). Efforts are already under way to further advance the YIF-SCAN system to analyze not only the *C. coccoides* group but also the other predominant groups; we aim to achieve a more comprehensive understanding of the microbiota in the context of factors influencing its composition and functions.

Several recent studies have suggested a link between the gut microbiota and host gender and sex hormones (Mueller et al., 2006; Li et al., 2008; Qin et al., 2010; Bolnick et al., 2014; Shastri et al., 2015; Haro et al., 2016; Oki et al., 2016). Prompted by this concept, we also analyzed our dataset of healthy Japanese young adults according to host gender. Interestingly, we came across several gender-specific differences. For instance, we found that females had higher counts of total bacteria, bifidobacteria, and *Lactobacillus gasseri* subgroup than their male counterparts (Suzuki et al., 2017). Furthermore, we noted several gender-specific differences in the association of gut microbiota with dietary habits, such as the frequency of yogurt consumption (Suzuki et al., 2017). Although the mechanisms underlying this association remain largely unclear, these gender-specific differences in intestinal bacterial carriage warrant further exploration.

## Features of the gut microbiota of asians and oceanians

Because RNA is more prone to degradation than DNA, the YIF-SCAN system requires the RNA content of samples to be maintained in fresh and RNase-free conditions. This has been achieved by treating the fecal specimen with the commercially available fixing agent RNA*later*. By using this method (which has already been validated), the microbial nucleic acids in the homogenized samples can be stably maintained (Kurakawa et al., 2012; Nomoto et al., 2015) under both refrigerated conditions and at ambient temperatures for up to 4 weeks, thereby making it possible to collect and preserve fecal samples, particularly in those settings where samples cannot be preserved at low temperature. This has enabled us to investigate the microbiota of not only Japanese populations but also people from other Asian countries. In one recent study (Nakayama et al., 2015), the gut microbiota of over 300 children from 10 cities in five Asian countries was investigated by simultaneously performing both the exhaustive analysis of 16S rRNA genes by pyrosequencing and the quantitative analysis of 16S rRNA by YIF-SCAN. The results revealed that the microbiota of these Asian children could be classified into two enterotype-like clusters: a *Bacteroides-* and *Bifidobacterium*-rich type and a *Prevotella*-rich type. Further investigation of the children's dietary and lifestyle factors revealed that the distribution of these two types was correlated with their intake of resistant starch. YIF-SCAN analysis has also revealed unique features of the gut microbiota of people living a subsistence lifestyle in Papua New Guinea (Greenhill et al., 2015). Papua New Guineans consume several starchy crops as their staple foods, and their total protein intake is limited. We can surmise that their microbiota has adapted to these nutritional features.

## Analyzing the gut microbiota and determining dysbiosis in disease states

The highly sensitive YIF-SCAN RT-qPCR detection system has enabled us to easily and precisely detect perturbations in the counts of predominant bacteria as well as LCCM bacteria in patients with diseases such as colorectal cancer, type 2 diabetes, major depressive disorder, anorexia nervosa, Parkinson's disease, inflammatory bowel disease, and ischemic stroke. The counts of the predominant obligate anaerobes and the concentrations of organic acids were significantly lower in colorectal cancer (CRC) patients than in healthy controls (Ohigashi et al., 2013). Among CRC patients with different Duke's stages there were no differences in the microbiota or in organic acid concentrations. Therefore, these changes are not the result of CRC progression but may be related to CRC onset (Ohigashi et al., 2013). YIF-SCAN analysis revealed for the first time the presence of gut dysbiosis and possible blood bacterial translocation, which is defined as the passage of viable indigenous bacteria from the gastrointestinal tract to extraintestinal sites, in Japanese type 2 diabetes (T2D) patients (Sato et al., 2014). Giving probiotic drinks to patients reduced bacterial translocation, and this reduction was accompanied by an improvement in the dysbiosis (Sato et al., 2017). Messaoudi et al. (2011) reported that the administration of probiotics, including *Bifidobacterium* and *Lactobacillus*, could contribute to the mental well-being of human subjects with low levels of stress. The results of YIF-SCAN analysis show that reduced counts of *Bifidobacterium* and *Lactobacillus* are more common in patients with major depressive disorder (MDD) than in healthy controls (Aizawa et al., 2016). Anorexia nervosa (AN) patients have significantly lower counts of total bacteria and predominant obligate anaerobes than healthy controls (Morita et al., 2015). Moreover, detailed analysis has detected *C. difficile* only in AN binge-eating/purging-subtype patients and not in either healthy subjects or AN restrictive patients (Morita et al., 2015). Cell counts of predominant anaerobic bacteria are significantly lower in Parkinson's disease (PD) patients than in healthy subjects (Hasegawa et al., 2015). A 2-year follow-up study by the same group recently revealed that lower counts of *Bifidobacterium* and *Bacteroides fragilis* in year 0 were associated with worsening on the Unified Parkinson's Disease Rating Scale over the next 2 years, suggesting that changes in the intestinal microbiota are associated with the progression of PD (Minato et al., 2017). Counts of *C. coccoides* group bacteria, such as *F. saccharivorans*, in active ulcerative colitis (UC) patients are lower than those in patients with quiescent UC or in healthy individuals (Takeshita et al., 2016). In a murine acute colitis model, a heat-killed preparation of a *F. saccharivorans* strain isolated from human feces markedly inhibited colonic inflammation (Takeshita et al., 2016). Multivariable linear regression analysis has revealed that ischemic stroke (IS) is independently associated with increased bacterial counts of *Atopobium* cluster and *Lactobacillus ruminis* subgroup. Furthermore, the changes in the prevalence of *L. ruminis* subgroup are positively correlated with changes in serum interleukin-6 levels, indicating that gut dysbiosis in IS patients is associated with host inflammation (Yamashiro et al., 2017).

These studies collectively demonstrate that the intestinal microbial cell counts of various predominant anaerobes are reduced in patients with mental or neurological disorders such as MDD (Aizawa et al., 2016), PD (Hasegawa et al., 2015), or AN (Morita et al., 2015), as well as in patients with CRC (Ohigashi et al., 2013), T2D (Sato et al., 2014), or IS (Yamashiro et al., 2017) (Table 3). In addition, gut dysbiosis has been reported in patients with inflammatory bowel disease (Takaishi et al., 2008; Carding et al., 2015; Takeshita et al., 2016) or other severe or critical illnesses (Hayakawa et al., 2011), as well as in patients undergoing surgery or chemotherapy (Aoki et al., 2012; Tanaka et al., 2012; Okazaki et al., 2013; Komatsu et al., 2016; Motoori et al., 2017) (Table 3). No doubt, such quantitative elucidations of the commonalities and specificities of dysbiosis in various disorders or during and after surgery are important in that they could help to reveal factors associated with the dysbiosis, facilitate disease diagnosis and prognostication, and improve therapeutic outcomes. Notably, reduced fecal concentrations of organic acids, probably because of reduced levels of predominant anaerobes, have been observed in some diseases (Takaishi et al., 2008; Ohigashi et al., 2013; Sato et al., 2014).

**Table 2:** Distributions of intestinal bacteria in healthy Japanese subjects aged 3 years or older (*n* = 1116).

**Bacterial group**	**Age groups**													
	**Y03**		**Y04–13**		**Y18–19**		**Y20–39**		**Y40–59**		**Y60–79**		**Y80 over**	
	**(*****n*** = **154)**		**(*****n*** = **118)**		**(*****n*** = **249)**		**(*****n*** = **165)**		**(*****n*** = **166)**		**(*****n*** = **162)**		**(*****n*** = **101)**	
	**Median^†^ [5%, 95%]**	**Modalty^‡^**	**Median [5%, 95%]**	**Modalty**	**Median [5%, 95%]**	**Modalty**	**Median [5%, 95%]**	**Modalty**	**Median [5%, 95%]**	**Modalty**	**Median [5%, 95%]**	**Modalty**	**Median [5%, 95%]**	**Modalty**
*C. coccoides* group	9.9 [9.2, 10.5]	Uni	9.9 [9.3, 10.5]	Uni	10.1 [9.4, 10.6]	Uni	10.0 [9.2, 10.6]	Uni	9.9 [9.1, 10.5]	Uni	9.9 [8.7, 10.7]	Uni	9.8 [9.0, 10.4]	Uni
*C. leptum* subgroup	10.2 [9.2, 10.9]	Uni	10.2 [9.5, 10.8]	Uni	9.9 [8.2, 10.5]	Uni	9.9 [8.6, 10.9]	Uni	10.0 [8.6, 10.6]	Uni	10.1 [8.9, 10.8]	Uni	10.1 [9.2, 10.9]	Uni
*B. fragilis* group	10.0 [9.1, 10.6]	Uni	9.6 [8.8, 10.3]	Uni	9.8 [8.6, 10.5]	Uni	9.8 [8.7, 10.7]	Uni	9.7 [8.7, 10.6]	Uni	9.65 [8.0, 10.5]	Uni	9.3 [8.1, 10.5]	Uni
*Bifidobacterium*	10.3 [8.9, 11.0]	Uni	10.4 [9.1, 11.3]	Uni	10.0 [6.9, 10.8]	Uni	10.0 [8.4, 10.9]	Uni	9.8 [7.2, 10.7]	Uni	9.4 [6.7, 10.5]	Uni	8.7 [5.7, 10.5]	Uni
*Atopobium* cluster	9.2 [8.2, 9.9]	Uni	9.0 [7.9, 10.0]	Uni	9.3 [7.7, 10.1]	Uni	9.5 [8.1, 10.1]	Uni	9.5 [8.1, 10.0]	Uni	9.5 [8.2, 10.1]	Uni	9.0 [8.5, 10.0]	Uni
*Prevotella*	6.3 [5.1, 8.2]	Uni	6.8 [5.1, 10.0]	Uni	7.3 [5.4, 10.4]	Multi^*^	6.6 [5.2, 10.1]	Uni	7.3 [5.2, 10.6]	Uni	7.5 [5.3, 10.6]	Multi^*^	6.5 [5.2, 9.7]	Uni
*C. perfringens*	5.00 [3.7, 7.3]	Uni	4.5 [3.0, 6.4]	Uni	4.7 [3.6, 7.1]	Uni	4.4 [2.4, 7.1]	Uni	4.8 [2.5, 7.1]	Uni	5.4 [3.0, 8.0]	Uni	4.9 [3.0, 8.8]	Uni
*Enterobacteriaceae*	7.8 [6.6, 8.6]	Uni	6.8 [5.4, 8.2]	Uni	6.9 [5.7, 8.4]	Uni	7.1 [5.5, 8.4]	Uni	7.0 [5.4, 8.9]	Uni	7.5 [5.7, 9.0]	Uni	8.2 [6.5, 9.4]	Uni
*Enterococcus*	7.6 [6.4, 8.8]	Uni	5.5 [3.4, 7.1]	Uni	6.1 [4.6, 8.3]	Uni	6.0 [4.3, 8.1]	Uni	5.9 [4.4, 8.1]	Uni	6.7 [4.6, 9.1]	Uni	7.5 [5.6, 9.5]	Uni
*Staphylococcus*	5.4 [4.2, 6.4]	Uni	5.0 [3.8, 6.1]	Uni	4.8 [3.6, 7.1]	Uni	5.0 [3.6, 6.7]	Uni	4.6 [3.2, 6.0]	Uni	4.9 [3.5, 6.2]	Uni	5.2 [4.2, 6.8]	Uni
*Lactobacillus*	6.3 [3.8, 8.3]	Uni	5.9 [3.3, 7.9]	Uni	5.8 [3.6, 7.7]	Uni	6.1 [4.2, 8.2]	Uni	6.5 [4.4, 8.1]	Uni	7.2 [4.8, 9.6]	Uni	7.5 [4.6, 9.9]	Uni

† *Bacterial count (log_10_ cells/g feces)*.

‡ Uni, unimodality; Multi, multimodality; as computed by using Hartigan's dip test statistics (

* *P < 0.05, significant multimodality)*.

**Table 3:** Features of gut dysbiosis in disease states or during and after treatment.

**Category**	**Disease**	**Number of subjects (patient/ healthy controls)**	**Characteristics of gut dysbiosis^a^**	**References**
Cancer	Colorectal cancer	49/93	Lower total bacterial count; lower count of major strict anaerobes and some facultative anaerobes	Ohigashi et al., 2013
	Gastric cancer	190/31	Lower count of *C. coccoides* group; higher count of *Enterobacteriaceae* and lactobacilli	Aoki et al., 2012
Inflammatory bowel disease	Ulcerative colitis	65/73	Lower total bacterial count; lower count of major strict anaerobes	Takaishi et al., 2008
	Ulcerative colitis	31/17/34^*^	Lower total bacterial count and lower count of major strict anaerobes, especially some genus of *Clostridium* cluster XIVa, in active UC patients vs. healthy controls and quiescent UC patients	Takeshita et al., 2016
	Crohn's disease	65/23	Lower total bacterial count; lower count of major strict anaerobes	Takaishi et al., 2008
Metabolic disease	Type 2 diabetes	50/50	Lower count of major strict anaerobes; higher count of lactobacilli	Sato et al., 2014
Nerve and mental diseases	Anorexia nervosa	21/25	Lower total bacterial count; lower count of major strict anaerobes and streptococci	Morita et al., 2015
	Major depressive disorder	57/43	Lower count of bifidobacteria and lactobacilli	Aizawa et al., 2016
	Parkinson's disease	36/52	Lower count of major strict anaerobes; higher count of lactobacilli	Hasegawa et al., 2015
	Ischemic stroke	41/40	Lower count of major strict anaerobes; higher count of enterococci	Yamashiro et al., 2017
Injury	Critical illness	15/12	Lower count of major strict anaerobes and lactobacilli	Hayakawa et al., 2011
**Category**	**Operation**	**Number of subjects (before/ after operation)**	**Characteristics of gut dysbiosis**^b^	**References**
Surgery	Laparoscopic colorectal surgery	97/97	Decrease in count of major strict anaerobes along with total bacterial count; increase in some facultative anaerobes	Komatsu et al., 2016
	Esophagectomy	34/34	Decrease in major strict anaerobes along with total bacterial count and increase in some facultative anaerobes	Tanaka et al., 2012
	Gastroenterological surgery	23/23	Increase in major facultative anaerobes such as *Enterobacteriaceae* and *Staphylococcus*	Okazaki et al., 2013
Chemotherapy	Neoadjuvant chemotherapy	27/31	Decrease in major strict anaerobes along with total bacterial count and increase in some facultative anaerobes^b^	Motoori et al., 2017

a *Patients vs. healthy controls*;

b *before vs. after surgery or chemotherapy*.

* *active ulcerative colitis / quiescent ulcerative colitis / healthy controls*.

## Quantifying the effects of probiotics on human gut microbial composition

Probiotics are defined as “live microorganisms which when administered in adequate amounts confer a health benefit on the host” (Reid, Food and Agricultural Organization of the United Nation and WHO, 2005; Hill et al., 2014). In Fuller's (1989) definition, which had been popular long before the introduction of this new definition, the main mechanism of action of probiotics was cited as “improving … intestinal microbial balance.” The current generalized definition fits more appropriately, in particular in the context of the ever-mounting evidence suggesting that a variety of mechanisms of action, including immune regulation, are at work. However, in essence, the original hypothesis that improving the intestinal microbiota—or, more specifically, normalizing the dysbiosis specific to various pathologic conditions—can effectively prevent or improve pathologies remains very solid. In this context, YIF-SCAN-based analysis has enabled us to quantitatively monitor the effects of probiotics on the composition of the gut microbiota. In a study of elderly residents of aged-care facilities, we have shown marked improvement of gut dysbiosis—such as a reduction in the levels of *Enterobacteriaceae* and indigenous pathobionts and an increase in the numbers of anaerobes usually predominant in the healthy adult gut—following continuous consumption of a probiotic beverage (a fermented milk product containing *Lactobacillus casei* strain Shirota) for 6 months (Nagata et al., 2016). In another study in healthy children (Wang et al., 2015), we have reported reduced levels of pathobionts (*Enterobacteriaceae* and *Staphylococcus*) and increased levels of beneficial bacterial groups (bifidobacteria and lactobacilli) after long-term continuous consumption of probiotics (the same probiotic beverage used in the above-mentioned study of elderly residents). In several other studies, too, we have normalized gut dysbiosis by probiotic administration to different cohorts of gastrectomized subjects and residents of aged-care facilities (Nagata et al., 2011; Aoki et al., 2014). Generally, the levels of indigenous pathobionts such as *C. perfringens* and *Pseudomonas* species are extremely low in feces, thereby making it difficult to precisely track their intestinal carriage. To this end, the high sensitivity and precision of RT-qPCR facilitate the analysis of the actual numbers of these pathobionts, thereby also potentially enabling us to control their levels. Indeed, this ability to analyze the intestinal microbiota over a wide dynamic range is very important for elucidating the enhancement of microbiota resilience by probiotics, as well as for identifying potential microbiota-based interventions to ameliorate disease.

## Applications in clinical diagnosis

From the viewpoint of clinical diagnosis, the precise and sensitive detection and enumeration of disease-causing organisms (which are generally present in low numbers in healthy individuals) becomes indispensable. In a study of patients with febrile neutropenia (Sakaguchi et al., 2010), we compared the use of the blood culture method with our RT-qPCR-based method for diagnosing bacteremia. Whereas the rate of detection of bacteria in the peripheral blood of patients was 17% with the blood culture method, YIF-SCAN analysis revealed bacteremia in as many as 70% of patients with fever and neutropenia. Also, in a comparison of the detection of sepsis by using YIF-SCAN and the conventional culture method, YIF-SCAN analysis yielded significantly greater detection rates than the blood culture method (Fujimori et al., 2010). In view of the fact that, for some pediatric patients for whom prompt treatment is required, therapy is sometimes started even before the blood culture results become available, the rapid and highly sensitive analytic ability of the RT-qPCR method could prove particularly useful in a clinical laboratory testing. We have also applied the system in the field of gastrointestinal surgery to investigate bacterial translocation during surgery (Mizuno et al., 2010; Yokoyama et al., 2014). Postoperative infectious complications due to long and complicated surgery, particularly in biliary tract cancer, are a major problem. We have investigated in detail the occurrence of bacterial translocation in patients with biliary tract cancer by examining the mesenteric lymph nodes at the beginning and end of surgery (Mizuno et al., 2010). Intestinal bacteria were detected in 29.4% of mesenteric lymph nodes at the beginning and 37.3% following surgery, suggesting that there was a marked increase in bacterial translocation. Further examination of bacterial gene sequences showed that the bacterial strains detected in the blood were the same as those detected in the intestine. In addition, the incidence of postoperative infectious complications was significantly higher in patients with bacterial translocation. These results clearly suggest that controlling bacterial translocation during surgery can play an important role in preventing postoperative infection. In a subsequent study, we showed that the perioperative use of synbiotics (combinations of probiotics and prebiotics) in patients with esophageal cancer could significantly reduce both bacterial translocation during surgery and the incidence of postoperative infectious complications (Yokoyama et al., 2014).

YIF-SCAN can be used not only for precise analysis of intestinal microbiota but also for sensitive laboratory testing. Culture methods are still typically employed in microbial diagnosis in clinical practice; however, in the case of difficult-to-culture and antibiotic-resistant pathogens (e.g., in the case of febrile neutropenia) it is difficult to arrive at a clear-cut diagnosis. In this context, we have been exploring the potential of YIF-SCAN in identifying pathogenic microbes. For example, selection pressure in the targeted culturing of *C. difficile*, the leading cause of antibiotic-associated diarrhea, markedly reduces quantification accuracy. By targeting 23S rRNA molecules, we have established a sensitive and accurate quantification system that can detect *C. difficile* even if its intestinal microbial cell counts are very low (Matsuda et al., 2012). Similarly, to facilitate the diagnosis of *Candida*, which is a common cause of mycosis and possesses species-specific differences in patterns of resistance to antimicrobial agents, we have validated a highly specific and sensitive quantification system for major pathogenic *Candida* species, including *Candida albicans, Candida glabrata, Candida tropicalis, Candida parapsilosis*, and *Candida krusei* (Ogata et al., 2015). With all of these attributes, YIF-SCAN can facilitate the rapid, efficient, and highly sensitive diagnosis of a wide variety of clinically problematic microbes.

The Human Microbiome Project is analyzing the bacterial gene content of the microbiota in a variety of body niches, including the gastrointestinal tract, oral cavity, skin, and vagina. In the context of the core microbiome concept, the importance of maintaining the stability of the bacterial microbiota of the intestine and vagina has been particularly emphasized (Huse et al., 2012). In this regard, YIF-SCAN has been proven to be an appropriate method for quantifying the various major bacterial groups constituting the vaginal microbiota across a wide range of bacterial numbers. These groups include *Lactobacillus* subgroups and species, as well as pathobionts such as *Gardnerella vaginalis* and *Atopobium vaginae* and pathogens causing sexually transmitted diseases. This YIF-SCAN is based on the analysis of RNA extracted from vaginal mucosal samples and is highly sensitive (Kurakawa et al., 2015b). The system has been used to examine the vaginal bacterial microbiota of healthy Japanese women. In examining the predominance of the *Lactobacillus* community, we noticed that the counts of several bacterial-vaginosis-associated bacteria were quite high in a number of asymptomatic subjects (Kurakawa et al., 2015b) This suggests that there is some sort of early-stage dysbiosis of the vaginal microbiota in the process of bacterial vaginosis. This finding needs to be clarified in further studies, and future research developments could make it possible to maintain a healthy vaginal microbiota as well as to prevent and improve various urogenital diseases by preventing dysbiosis.

## Conclusion and prospects

This novel approach to bacterial enumeration using rRNA-molecule-targeting RT-qPCR has been remarkably helpful in quantifying the dynamic changes occurring in the gut microbiota during host aging, especially during infancy and early childhood. By using a large integrated database of healthy Japanese people, we have revealed that not only the predominant obligate anaerobes but also facultative anaerobes constitute the core microbiota, and that the counts of each bacterial group approximate a log-normal distribution. We are sure that the information generated from this large integrated cohort will be helpful in further elucidating the features of the gut microbiota in prospective studies. All in all, microbial investigations using YIF-SCAN-based analyses have increased our knowledge of the structure, function, and dynamics of the complex microbial ecosystem both in health and in disease. The ultimate goal is to capitalize on this knowledge to improve human health. We are convinced that expanding the detection range of this analytical system to more microbial clades will provide a more comprehensive picture of the microbiota. Certainly, the scope of this valuable analytical tool will be realized in settings beyond the gastrointestinal tract, and its range of applications will broaden to cover various other niches of the human body and the microbiota of other host species in other biological settings.

## Author contributions

HT analyzed data. HT and KN wrote manuscript. HT, KM, and KN checked and revised manuscript. HT, KM, and KN approved final version of manuscript.

### Conflict of interest statement

The authors declare that the research was conducted in the absence of any commercial or financial relationships that could be construed as a potential conflict of interest.
