# Supplementary material for: Counting the Countless: Bacterial Quantification by Targeting rRNA Molecules to Explore the Human Gut Microbiota in Health and Disease
Source: Front Microbiol. 2018 Jun 29;9:1417. doi: 10.3389/fmicb.2018.01417 (PMC6033970; doi:10.3389/fmicb.2018.01417)
Supplement: Supplementary file 1 [file Table_1.docx]

***Supplementary Material***

Counting the countless: Targeting rRNA molecules for bacterial quantification: a novel and potential approach for exploring human gut microbiota in health and disease.

Hirokazu Tsuji*, Kazunori Matsuda, Koji Nomoto

***Correspondence:** Hirokazu Tsuji, PhD: [hirokazu-tsuji@yakult.co.jp](mailto:hirokazu-tsuji@yakult.co.jp)

**Supplementary Table 1.** General information of the subjects included in the healthy Japanese intestinal microbiota database.

| Age group | Average age  (mean ± SD) | No. of volunteers | | Reference |
| --- | --- | --- | --- | --- |
|  |  | Total | Men/Women |  |
| 1 day | 1.0 ± 0.3 days | 151 | 82/69 | *Tsuji et al., 2012* |
| 3 days | 3.0 ± 0.2 days | 149 | 79/70 |  |
| 7 days | 7.2 ± 1.3 days | 146 | 82/64 |  |
| 1 month | 0.9 ± 0.1 months | 146 | 80/66 |  |
| 3 months | 2.7 ± 0.1 months | 121 | 63/58 |  |
| 6 months | 5.5 ± 0.1 months | 123 | 70/53 |  |
| 3 years | 3.0 ± 0.1 years | 154 | 82/72 |  |
| 4-13 years | 8.7 ± 1.5 years | 118 | 63/55 | *Nakayama et al., 2015;* *Wang et al., 2015* |
| 18-19 years | 19.1 ± 0.3 years | 249 | 183 / 66 | *Suzuki et al., 2017* |
| 20‒39 years | 28.0 ± 6.6 ± 5.5 years | 165 | 63 / 102 | *Aizawa et al., 2016; Aoki et al., 2014; Bian et al., 2011; Hasegawa et al., 2015; Kanda, 2013; Matsuda et al., 2009; Morita et al., 2015; Nagata et al., 2011, 2016; Ohigashi et al., 2013; Sato et al., 2014; Tsuji et al., 2014)* |
| 40‒59 years | 49.7 ± 5.8 years | 166 | 41/125 |  |
| 60‒79 years | 68.6 ± 5.5 years | 162 | 75/87 |  |
| 80 years or more | 87.4 ± 5.2 years | 101 | 25/76 |  |
| Sum | | 1951 | 988/963 |  |

**Supplementary Materials and methods**

Subjects

We used the data of intestinal bacterial microbiota from healthy subjects enrolled in different studies (n=1,951) wherein bacterial counts and prevalence was analyzed by using YIF-SCAN (Supplementary Table 1). In case of studies where any probiotics intervention was involved, the data from only the before-intake period was used for the present analysis.

Target bacteria

To analyze the intestinal bacterial microbiota, the following bacterial groups, genera, and species were quantified: *Clostridium coccoides* group, *Clostridium leptum* subgroup, *Bacteroides fragilis* group, *Bifidobacterium*, *Atopobium* cluster, *Prevotella*, Enterobacteriaceae, *Enterococcus*, *Staphylococcus*, *Lactobacillus*, and *Clostridium perfringens.* The total bacterial count was expressed as the sum of bacterial counts of all the bacterial groups, genera and species analyzed. The *Lactobacillus* count was calculated as the sum of bacterial counts of six subgroups (*Lactobacillus casei* subgroup, *Lactobacillus gasseri* subgroup, *Lactobacillus plantarum* subgroup, *Lactobacillus reuteri* subgroup, *Lactobacillus ruminis* subgroup, and *Lactobacillus sakei* subgroup) and two species (*Lactobacillus brevis*, *Lactobacillus fermentum*).

Statistical analysis

R version 3.3.1. and EZR 1.33 (Kanda, 2013) were used for an exploratory analysis of the integrated microbiota data. For principal component analysis (PCA), if the data showed values below the detection limit, one-half of the detection limit value for each bacterial group, genus, or species was used as the input value. We used GMD program operating with R language for hierarchical clustering and heatmap analysis; ade4 or ggplot2 program for PCA analysis; and ggplot2 program for histograms, dot plots and box plots. The Hartigans' dip test statistic (diptest R package) was used to compute unimodality/ multimodality of the fecal bacterial counts, wherein *P* value less than 0.05 indicated significant multimodality.

**References**

Aizawa, E., Tsuji, H., Asahara, T., Takahashi, T., Teraishi, T., Yoshida, S., et al. (2016). Possible association of *Bifidobacterium* and *Lactobacillus* in the gut microbiota of patients with major depressive disorder. *J. Affect. Disord.* 202, 254–257. doi:10.1016/j.jad.2016.05.038.

Aoki, T., Asahara, T., Matsumoto, K., Takada, T., Chonan, O., Nakamori, K., et al. (2014). Effects of the continuous intake of a milk drink containing *Lactobacillus casei* strain Shirota on abdominal symptoms, fecal microbiota, and metabolites in gastrectomized subjects. *Scand. J. Gastroenterol.* 49, 552–63. doi:10.3109/00365521.2013.848469.

Bian, L., Nagata, S., Asahara, T., Rahman, M. S., Ohta, T., Yuki, N., et al. (2011). Effects of the continuous intake of *Lactobacillus casei* strain shirota- fermented milk on risk management of long-term inpatients at health. *Int. J. Probiotics Prebiotics* 6, 123–132.

Hasegawa, S., Goto, S., Tsuji, H., Okuno, T., Asahara, T., Nomoto, K., et al. (2015). Intestinal dysbiosis and lowered serum lipopolysaccharide-binding protein in parkinson’s disease. *PLoS One* 10, e0142164. doi:10.1371/journal.pone.0142164.

Kanda, Y. (2013). Investigation of the freely available easy-to-use software “EZR” for medical statistics. *Bone Marrow Transplant.* 48, 452–8. doi:10.1038/bmt.2012.244.

Matsuda, K., Tsuji, H., Asahara, T., Matsumoto, K., Takada, T., and Nomoto, K. (2009). Establishment of an analytical system for the human fecal microbiota, based on reverse transcription-quantitative PCR targeting of multicopy rRNA molecules. *Appl. Environ. Microbiol.* 75, 1961–9. doi:10.1128/AEM.01843-08.

Morita, C., Tsuji, H., Hata, T., Gondo, M., Takakura, S., Kawai, K., et al. (2015). Gut Dysbiosis in Patients with Anorexia Nervosa. *PLoS One* 10, e0145274. doi:10.1371/journal.pone.0145274.

Nagata, S., Asahara, T., Ohta, T., Yamada, T., Kondo, S., Bian, L., et al. (2011). Effect of the continuous intake of probiotic-fermented milk containing *Lactobacillus casei* strain Shirota on fever in a mass outbreak of norovirus gastroenteritis and the faecal microflora in a health service facility for the aged. *Br. J. Nutr.* 106, 549–56. doi:10.1017/S000711451100064X.

Nagata, S., Asahara, T., Wang, C., Suyama, Y., Chonan, O., Takano, K., et al. (2016). The Effectiveness of Lactobacillus Beverages in Controlling Infections among the Residents of an Aged Care Facility: A Randomized Placebo-Controlled Double-Blind Trial. *Ann. Nutr. Metab.* 68, 51–9. doi:10.1159/000442305.

Nakayama, J., Watanabe, K., Jiang, J., Matsuda, K., Chao, S.-H., Haryono, P., et al. (2015). Diversity in gut bacterial community of school-age children in Asia. *Sci. Rep.* 5, 8397. doi:10.1038/srep08397.

Ohigashi, S., Sudo, K., Kobayashi, D., Takahashi, O., Takahashi, T., Asahara, T., et al. (2013). Changes of the intestinal microbiota, short chain fatty acids, and fecal pH in patients with colorectal cancer. *Dig. Dis. Sci.* 58, 1717–26. doi:10.1007/s10620-012-2526-4.

Sato, J., Kanazawa, A., Ikeda, F., Yoshihara, T., Goto, H., Abe, H., et al. (2014). Gut dysbiosis and detection of “live gut bacteria” in blood of Japanese patients with type 2 diabetes. *Diabetes Care* 37, 2343–50. doi:10.2337/dc13-2817.

Suzuki, Y., Ikeda, K., Sakuma, K., Kawai, S., Sawaki, K., Asahara, T., et al. (2017). Association between Yogurt Consumption and Intestinal Microbiota in Healthy Young Adults Differs by Host Gender. *Front. Microbiol.* 8, 847. doi:10.3389/fmicb.2017.00847.

Tsuji, H., Chonan, O., Suyama, Y., Kado, Y., Nomoto, K., Nanno, M., et al. (2014). Maintenance of healthy intestinal microbiota in women who regularly consume probiotics. *Int. J. Probiotics Prebiotics* 9, 31–38.

Tsuji, H., Oozeer, R., Matsuda, K., Matsuki, T., Ohta, T., Nomoto, K., et al. (2012). Molecular monitoring of the development of intestinal microbiota in Japanese infants. *Benef. Microbes* 3, 113–25. doi:10.3920/BM2011.0038.

Wang, C., Nagata, S., Asahara, T., Yuki, N., Matsuda, K., Tsuji, H., et al. (2015). Intestinal microbiota profiles of healthy pre-school and school-age children and effects of probiotic supplementation. *Ann. Nutr. Metab.* 67, 257–266. doi:10.1159/000441066.
